# ﻿Unveiling species diversity within early-diverging fungi from China II: Three new species of *Absidia* (Cunninghamellaceae, Mucoromycota) from Hainan Province

**DOI:** 10.3897/mycokeys.110.129120

**Published:** 2024-11-20

**Authors:** Meng-Fei Tao, Zi-Ying Ding, Yi-Xin Wang, Zhao-Xue Zhang, Heng Zhao, Zhe Meng, Xiao-Yong Liu

**Affiliations:** 1 College of Life Sciences, Shandong Normal University, Jinan, 250358, China Shandong Normal University Jinan China; 2 Shandong Provincial Key Laboratory for Biology of Vegetable Diseases and Insect Pests, College of Plant Protection, Shandong Agricultural University, Taian, 271018, China Shandong Agricultural University Taian China; 3 School of Ecology and Nature Conservation, Beijing Forestry University, Beijing 100081, China Beijing Forestry University Beijing China; 4 State Key Laboratory of Mycology, Institute of Microbiology, Chinese Academy of Sciences, Beijing 100101, China Institute of Microbiology, Chinese Academy of Sciences Beijing China

**Keywords:** Basal fungi, fungal diversity, molecular phylogeny, Mucorales taxonomy

## Abstract

*Absidia* is distributed worldwide and primarily isolated from soil, feces, and decaying plants. The genus was initially classified into Absidiaceae and then Mucoraceae, and currently belongs to Cunninghamellaceae and is further divided into *Absidia* s.s., *Lichtheimia*, and *Lentamyces*. Three new species of *Absidia* s.s. are identified and described from soil in Hainan Province of China based on morphological characteristics, molecular data, and maximum growth temperatures as well. They are named based on distinct shapes of projections on columellae: *A.crystalloides***sp. nov.** (crystal-like), *A.pacifica***sp. nov.** (pacifier-like), *A.pateriformis***sp. nov.** (bowling-like). In SSU-ITS-LSU-TEF-*Act* phylogram, the *A.crystalloides* is closely related to *A.oblongispora* and *A.heterospora*, the *A.pacifica* is a sister group with *A.edaphica*, and the *A.pateriformis* has a close relationship with *A.jiangxiensis*. This study enriches the species diversity of *Absidia* in China.

## ﻿Introduction

The genus *Absidia* Tiegh. 1878 belongs to Mucoromycota, Mucoromycetes, *Mucorales*, Cunninghamellaceae (http://www.indexfungorum.org/, accessed on 15 March 2024). The type species of this genus is *A.reflexa* Tiegh. 1878 (van Tieghem 1876). Its classification has been a subject of controversy for over a century. *Absidia* was initially classified in the family Absidiaceae (von Arx 1982) and later in the family Mucoraceae (Benny et al. 2001). Eventually, it was included in the family Cunninghamellaceae (Ashton 2009). Recently, *Absidia* s.l. was divided into *Absidia* s.s., *Lichtheimia* Vuill, and *Lentamyces* Kerst. Hoffm. & K. Voigt based on growth temperature, molecular phylogeny, and morphological characteristics (Hoffmann et al. 2007; Hoffmann and Voigt 2009; Hoffmann 2010). The *Absidia* is distributed globally, with reports of its presence in Estonia (1621), Australia (839), Czechia (688), Argentina (514), and Lithuania (422) (https://www.gbif.org/, accessed on 15 March 2024). The genus is primarily found in soil, but it can also be detected in feces, air, decaying plants, and waste materials (Zhang et al. 2018; Lima et al. 2020; Zong et al. 2021; Hurdeal et al. 2021, 2023). Currently, there are 131 records of *Absidia* species, varieties and subspecies (http://www.indexfungorum.org/, accessed on 15 March 2024). Zhao et al. (2023) discovered 15 novel *Absidia* species from Beijing, Yunnan, Qinghai, and Xinjiang of China, along with three Chinese new record species. This significant contribution has substantially augmented the Chinese database on *Absidia* species resources and enhanced the taxonomic framework of Mucoromycota (Zhao et al. 2023). *Absidia* is frequently employed in the biotransformation of various types of natural products, including hydroxylation, glycosylation, reduction reactions, and more. Both *A.caerulea* and *A.glauca* can be utilized for the biotransformation of flavones and flavanones (Sordon et al. 2019). *A.spinosa* is applied in the biotransformation of cresol red (Kristanti et al. 2016). Additionally, it should be noted that species in those genera closely related to *Absidia* plays a vital role as a pathogen causing human mucormycosis (Constantinides et al. 2008); specifically, *A.corymbifera* (=*Lichtheimiacorymbifera*) is associated with meningitis (Mackenzie et al. 1988) and keratitis (Varona et al. 2015). The typical characteristics of *Absidia* include stolons, rhizoids, sporangia, sporangiophores, etc. The sporangiophores can be branched or unbranched and may grow erect or curved. If a collar is present, it is easily noticeable. One or two protrusions are present on columellae. It produces zygospores that are typically spherical in shape (van Tieghem 1876; Hoffmann et al. 2007; Zong et al. 2021; Zhao et al. 2023).

While investigating soil microbial resources in Hainan Province, three novel *Absidia* species (*A.crystalloides* sp. nov., *A.pacifica* sp. nov., and *A.pateriformis* sp. nov.) were discovered. These new species are illustrated in this article based on morphological features, molecular phylogeny, and maximum growth temperatures.

## ﻿Materials and methods

### ﻿Sample collection and strain isolation

The strains were isolated from soil samples collected from Jianfengling Mountains and Bawangling Mountains in Hainan Province in October 2023. Samples were subjected to the moist-chamber culture and plate dilution coating methods (Zhao et al. 2022a, 2022b). The moist-chamber culture involved evenly sprinkling 1 g of soil onto the surface of PDA plates (potato 200 g, dextrose 20 g, agar 20 g, distilled water 1000 mL), sealing with a parafilm, inverting the plates, and incubating at 25 °C in darkness. After three days, microscopic observation was conducted, and the target strain was purified using an inoculating loop upon the formation of sporangia of *Absidia*. The plate dilution coating included taking 1 g of soil, diluting with 1 mL of sterile water through swirling and shaking until reaching dilutions of 10^-2^, 10^-3^, and 10^-4^. Subsequently, 200 µL of the diluted solutions (10^-3^ and 10^-4^) were evenly applied onto the surface of PDA plates using sterile triangle spreaders. The plates were sealed with a parafilm and inverted for cultivation at 25 °C in darkness. After three days, purification of target strains was performed. The target strain was stored in four freezing tubes and preserved in a refrigerator at a temperature of 4 °C. The purified strains were preserved in the China General Microbiological Culture Collection Center, Beijing, China (CGMCC). Additionally, they were stored in the Shandong Agricultural University Culture Collection (SAUCC) and Shandong Normal University, Jinan, China (XG). The dried cultures were maintained in the Herbarium Mycologicum Academiae Sinicae, Beijing, China (HMAS).

### ﻿Morphology and maximum growth temperature

The PDA cultured colonies were incubated at a temperature of 25 °C, and on the seventh day of colony growth, photographs were captured from both the front and back using a digital camera (Canon Powershot G7X). The colonies were allowed to grow for a period of five to six days, and then their microscopic morphology was observed using Olympus SZX10 stereomicroscope and Olympus BX53 microscope. Morphological features were assessed by measuring 20 variables, including minimum and maximum values. Two strains were inoculated on PDA containing 0.1% lecithin for pairing experiments, and the petri dishes were sealed with parafilm to maintain moisture. To determine the maximum growth temperature, a gradient method (Zheng et al. 2007, 2009; Zheng and Liu 2009; Zong et al. 2021; Zhao et al. 2022b) was employed whereby the colonies were initially cultured at 25 °C for two days followed by an increase in temperature by 1 °C each day until no further growth was observed. This determined the maximum temperature. The species were classified and described, and the taxonomic information was uploaded to Fungal Name repository (https://nmdc.cn/fungalnames/).

### ﻿DNA extraction, PCR amplification, and sequencing

In this study, the CTAB method (Doyle and Doyle 1990; Guo et al. 2000) was employed for DNA extraction. Subsequently, the extracted DNA underwent polymerase chain reaction (PCR) targeting five segments, namely SSU (small subunit), ITS (internal transcribed spacer), LSU (large subunit of ribosomal DNA), TEF-1α (translation elongation factor 1 alpha), and *Act* (actin). For PCR amplification, five primer pairs were utilized: NS1/NS4, ITS4/ITS5, LR0R/LR5, EF1-983F/TEF1LLErev, and Act-1/Act-4R (Table 1). The amplification reaction solution (25 μL) contained 12.5 μL 2× Hieff Canace Plus PCR Master Mix with dye (Yeasen Biotechnology, Shanghai, China, Cat No. 10154ES03), 9.5 μL ddH_2_O, 1 μL forward primer (10 μM), 1 μL reverse primer (10 μM) and 1 μL DNA template (1 ng/μL). The PCR programs are listed in Table 1. Following PCR amplification, the products were subjected to electrophoresis using 2% agarose gels and observed under UV light to confirm successful amplification (Zhang et al. 2021). Amplified products were purified through recovery using a Gel Extraction Kit (Cat# AE0101-C, Shandong Sparkjade Biotechnology Co., Ltd.). Tsingke Biotechnology Co., Ltd. (Qingdao, China) was responsible for DNA sequencing and primer synthesis. MEGA v. 7.0 (Kumar et al. 2016) was employed for sequence assembly. Finally, the sequence was compared against NCBI (National Center for Biotechnology Information) database to search relatives.

**Table 1.** Experimental condition of PCR used in this study.

Loci	PCR primers	Primer sequence (5’ – 3’)	PCR cycles	References
SSU	NS1; NS4	GTA GTC ATA TGC TTG TCT C C; CTT CCG TCA ATT CCT TTA AG	95 °C 5 min; (94 °C 60 s, 54 °C 50 s, 72 °C 60 s) × 37 cycles; 72 °C 10 min	(White et al. 1990)
ITS	ITS5; ITS4	GGA AGT AAA AGT CGT AAC AAG G; TCC TCC GCT TAT TGA TAT GC	95 °C 5 min; (95 °C 30 s, 55 °C 30 s, 72 °C 1 min) × 35 cycles; 72 °C 10 min	(White et al. 1990)
LSU	LR0R; LR5	GTA CCC GCT GAA CTT AAG C; TCC TGA GGG AAA CTT CG	95 °C 5 min; (94 °C 30 s, 52 °C 45 s, 72 °C 90 s) × 30 cycles; 72 °C 10 min	(Hurdeal et al. 2023)
TEF-1α	EF1-983F; TEF1LLErev	GCYCCYGGHCAYCGTGAYTTYAT; AACTTGCAGGCAATGTGG	95 °C 5 min; (95 °C 30 s, 55 °C 60 s,72 °C 60 s) × 30 cycles; 72 °C 10 min	(Rehner and Buckley 2005; Jaklitsch et al. 2005)
*Act*	Act-1; Act-4R	TGG GAC GAT ATG GAI AAI ATC TGG CA; TC ITC GTA TIC TIG CTI IGA IAT CCA CA T	95 °C 3 min; (95 °C 60 s, 55 °C 60 s,72 °C 60 s) × 30 cycles; 72 °C 10 min	(Voigt and Wöstemeyer 2000)

### ﻿Phylogenetic analyses

Referring to the newly published article about *Absidia* (Zhao et al. 2023), the sequences were downloaded for phylogenetic analysis from the NCBI (https://www.ncbi.nlm.nih.gov/), and the GenBank accession numbers of the sequences used are shown in Table 2. All sequences were compared and manually corrected using MEGA v.7.0 (Kumar et al. 2016). The phylogeny of *Absidia* was inferred using the maximum likelihood (ML), Bayesian inference (BI), and maximum parsimony (MP) algorithms. ML analysis was conducted using RaxML 8.2.4 in CIPRES Science Gateway V. 3.3 (https://www.phylo.org/), with 1,000 bootstrap replicates (Miller et al. 2010; Stamatakis 2014). Eight cold Markov chains ran simultaneously for two million generations using the GTR + I + G model for BI analysis, and sampling once every 1,000 generations (Huelsenbeck and Ronquist 2001; Nylander 2004). MP analysis was performed for 1,000 bootstrap replicates by the heuristic search option with reconnection and bisection (Swofford 2002). The result of phylogenetic analysis was checked and adjusted using FigTree v.1.4.4 (http://tree.bio.ed.ac.uk/software/figtree), followed by beautifying the phylogenetic tree with Adobe Illustrator CC 2019. Base differences among the three new species and related species were calculated with MEGA7, with evolutionary distances and standard deviations being shown below and above the diagonal line, respectively. (Kumar et al. 2016).

**Table 2.** GenBank accession numbers of sequences used in this study.

Species	Strains	GenBank accession numbers				
		ITS	LSU	TEF-1α	Act	SSU
*Absidiaabundans*	XY09265	ON074697	ON074681	NA	NA	NA
*A.abundans*	CGMCC 3.16255*	NR_182590	ON074683	NA	NA	NA
*A.abundans*	XY09274	ON074696	ON074682	NA	NA	NA
*A.aguabelensis*	URM 8213*	NR_189383	NG_241934	NA	NA	NA
*A.alpina*	CGMCC 3.16104	OL678133	NA	NA	NA	NA
*A.ampullacea*	CGMCC 3.16054	MZ354138	MZ350132	NA	NA	NA
*A.anomala*	CBS 125.68*	MH859085	MH870799	NA	NA	NA
*A.anomala*	FSU5798	EF030523	NA	NA	EF030535	NA
*A.biappendiculata*	CBS 187.64	MZ354153	MZ350147	MZ357420	MZ357438	NA
*A.bonitoensis*	URM 7889*	MN977786	MN977805	NA	NA	NA
*A.brunnea*	CGMCC 3.16055*	MZ354139	MZ350133	MZ357403	MZ357421	NA
*A.caatinguensis*	URM 7156*	NR_154704	NG_058582	NA	NA	NA
*A.caerulea*	XY00608	OL620081	NA	NA	NA	NA
*A.caerulea*	XY00729	OL620082	NA	NA	NA	NA
*A.caerulea*	CBS101.36	MH855718	MH867230	NA	NA	NA
*A.caerulea*	FSU767	AY944870	NA	NA	NA	NA
*A.californica*	CBS 314.78	JN205816	MH872902	NA	NA	NA
*A.californica*	FSU4748	AY944873	EU736301	EU736247	EU736224	EU736274
*A.californica*	FSU4747	AY944872	EU736300	EU736246	AY944758	EU736273
*A.chinensis*	CGMCC 3.16057	MZ354141	MZ350135	NA	MZ357422	NA
*A.chinensis*	CGMCC 3.16056*	MZ354140	MZ350134	NA	NA	NA
*A.cinerea*	CGMCC 3.16062	MZ354146	MZ350140	MZ357407	MZ357427	NA
*A.cornuta*	URM 6100*	NR_172976	MN625255	NA	NA	NA
***A.crystalloides***	CGMCC3.27496*	**PP377803**	**PP373736**	**PP790574**	**PP790582**	**PP779723**
***A.crystalloides***	**SAUCC693201**	**PP377804**	**PP373737**	**PP790573**	**PP790581**	**PP779722**
*A.cuneospora*	CBS 101.59*	MH857828	MH869361	NA	NA	NA
*A.cylindrospora*	CBS 100.08	JN205822	JN206588	NA	NA	NA
*A.digitula*	CGMCC 3.16058*	MZ354142	MZ350136	MZ357404	MZ357423	NA
*A.edaphica*	MFLUCC 20-0088	NR_172305	NG_075367	NA	MT410739	NG_074951
*A.frigida*	CGMCC 3.16201*	NR_182565	OM030223	NA	NA	NA
*A.fusca*	CBS 102.35*	NR_103625	NG_058552	NA	NA	NA
*A.gemella*	CGMCC 3.16202*	OM108488	OM030224	NA	NA	NA
*A.glauca*	CBS 129233	MH865253	MH876693	NA	NA	NA
*A.glauca*	CBS 101.08*	MH854573	MH866105	NA	NA	NA
*A.glauca*	FSU660	AY944879	EU736302	EU736248	EU736225	EU736275
*A.globospora*	CGMCC 3.16031*	NR_189829	MW671544	MZ357412	MZ357431	NA
*A.globospora*	CGMCC 3.16035	MW671538	MW671545	MZ357413	MZ357432	NA
*A.globospora*	CGMCC 3.16036	MW671539	MW671546	MZ357414	MZ357433	NA
*A.heterospora*	SHTH021	JN942683	JN982936	NA	NA	JQ004928
*A.jiangxiensis*	CGMCC 3.16105*	OL678134	PP780377	PP790569	PP790577	PP779719
*A.jindoensis*	CNUFC-PTI1-1	MF926622	MF926616	MF926513	MF926510	MF926626
*A.koreana*	EML-IFS45-1*	KR030062	KR030056	KR030060	KR030058	KT321298
*A.koreana*	XY00816	OL620083	ON123771	NA	NA	NA
*A.koreana*	XY00596	OL620084	NA	NA	NA	NA
*A.lobata*	CGMCC 3.16256	ON074690	ON074679	NA	NA	NA
*A.longissima*	CGMCC 3.16203*	NR_182566	OM030225	NA	NA	NA
*A.macrospora*	FSU4746	AY944882	EU736303	EU736249	AY944760	EU736276
*A.medulla*	CGMCC 3.16034	NR_189832	MW671549	MZ357417	MZ357436	NA
*A.montepascoalis*	URM 8218	NR_172995	NA	NA	NA	NA
*A.multispora*	URM 8210*	MN953780	MN953782	NA	NA	NA
*A.nigra*	CBS 127.68*	NR_173068	MZ350146	MZ357419	MZ357437	NA
*A.nigra*	CGMCC 3.16059	MZ354143	MZ350137	MZ357405	MZ357424	NA
*A.nigra*	CGMCC 3.16060	MZ354144	MZ350138	MZ357406	MZ357425	NA
*A.oblongispora*	CGMCC 3.16061	MZ354145	MZ350139	NA	MZ357426	NA
*A.ovalispora*	CGMCC 3.16019	NR_176748	MW264131	NA	NA	NA
***A.pacifica***	CGMCC3.27497*	**PP377802**	**PP373735**	**PP839793**	**PP790579**	**PP779720**
***A.pacifica***	**SAUCC413601**	**PP377801**	**PP373734**	**PP839794**	**PP790580**	**PP779721**
*A.panacisoli*	SYPF 7183*	MF522181	MF522180	MF624251	NA	MF522179
*A.pararepens*	XY00631	OL620085	ON123774	NA	NA	NA
*A.pararepens*	XY00615	OL620086	NA	NA	NA	NA
*A.pararepens*	XY05899	OL620087	NA	NA	NA	NA
*A.pararepens*	CCF 6352	MT193669	MT192308	NA	NA	NA
***A.pateriformis***	CGMCC3.27495*	**PP377805**	**PP373738**	**PP790575**	**PP790583**	**PP779724**
***A.pateriformis***	**SAUCC634702**	**PP377806**	**PP373739**	**PP790576**	**PP790584**	**PP779725**
*A.pernambucoensis*	URM<BRA>7219	MN635568	MN635569	NA	NA	NA
*A.pseudocylindrospora*	EML-FSDY6-2	KU923817	KU923814	NA	KU923815	KU923819
*A.psychrophilia*	FSU4745	AY944874	EU736306	EU736252	AY944762	EU736279
*A.purpurea*	CGMCC 3.16106	OL678135	NA	NA	NA	NA
*A.radiata*	CGMCC 3.16257	ON074698	ON074684	NA	NA	NA
*A.radiata*	XY09330-1	ON074699	ON074685	NA	NA	NA
*A.repens*	CBS 115583*	NR_103624	NG_058551	NA	NA	NA
*A.saloaensis*	URM 8209*	MN953781	MN953783	NA	NA	NA
*A.sichuanensis*	CGMCC 3.16258*	NR_182589	ON074688	NA	NA	NA
*A.soli*	MFLU-20-0414*	MT396373	MT393988	NA	NA	MT394049
*A.spinosa*	FSU551	AY944887	EU736307	EU736253	EU736227	EU736280
*A.stercoraria*	EML-DG8-1*	KU168828	KT921998	KT922002	KT922000	NG_065640
*A.sympodialis*	CGMCC 3.16063*	MZ354147	MZ350141	NA	NA	NA
*A.sympodialis*	CGMCC 3.16064	MZ354148	MZ350142	MZ357408	NA	NA
*A.terrestris*	FMR 14989*	LT795003	LT795005	NA	NA	NA
*A.turgida*	CGMCC 3.16032*	NR_189830	NG_241931	MZ357415	MZ357434	NA
*A.varians*	CGMCC 3.16065*	MZ354149	MZ350143	MZ357409	MZ357428	NA
*A.virescens*	CGMCC 3.16066*	MZ354150	MZ350144	MZ357410	MZ357429	NA
*A.virescens*	CGMCC 3.16067	MZ354151	MZ350145	MZ357411	MZ357430	NA
*A.xinjiangensis*	CGMCC 3.16107*	OL678136	NA	NA	NA	NA
*A.yunnanensis*	XY09528	ON074701	ON074686	NA	NA	NA
*A.yunnanensis*	CGMCC 3.16259*	NR_182591	NG_149054	NA	NA	NA
*A.zonata*	CGMCC 3.16033*	NR_189831	MW671548	MZ357416	MZ357435	NA
*A.zygospora*	RSPG 214	KC478527	NA	NA	NA	NA
*A.zygospora*	ANG28	DQ914420	NA	NA	NA	NA
*Cunninghamellablakesleeana*	CBS 782.68	JN205869	MH870950	NA	NA	NA
*C.elegans*	CBS 167.53	MH857146	HM849700	NA	NA	NA

Notes: The newly discovered species identified in the study are in bold. Ex-type strains are marked with a “^*^”. NA stands for “not available”.

## ﻿Result

### ﻿Phylogeny

**Figure 1.**
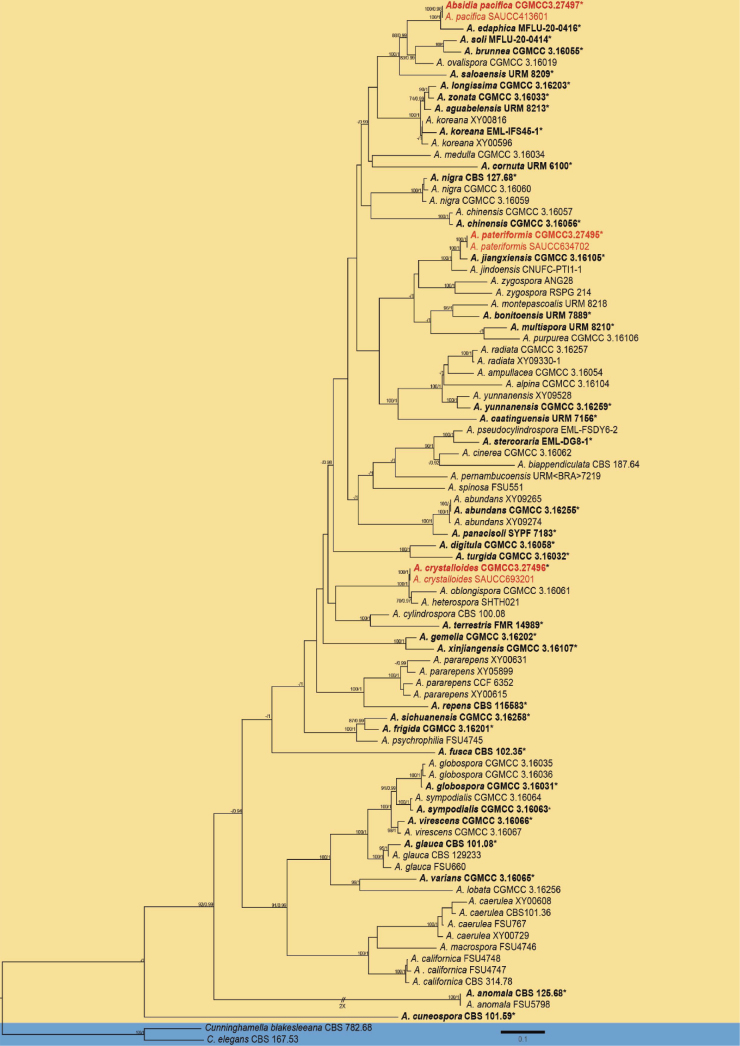
The maximum likelihood phylogram of *Absidia* based on SSU, ITS, LSU rDNA, TEF-1α and *Act* sequences with *Cunninghamellablakesleeana and C.elegans* as outgroups. The branches are marked with the Maximum Likelihood Bootstrap Value (left, MLBV ≥ 70%) and Bayesian Inference Posterior Probability (right, BIPP ≥ 0.90) at nodes. The six newly identified strains are denoted in red. Ex-type or ex-epitype strains are indicated with “*”.

Phylogenetic analyses of SSU, ITS, LSU rDNA, TEF-1α and *Act* were conducted for a total of 91 sequences, composing 36 species of *Absidia* and two outgroups, *Cunninghamellablakesleeana* (CBS 782.68) and *C.elegans* (CBS 167.53). The phylogenetic analysis encompassed a total of 4,785 characters, with 516 of SSU, 996 of ITS, 1,874 of LSU, 743 of TEF-1α, 660 of *Act* characters (Suppl. material 2). Among these, there were 2,712 constant and 515 variable but parsimony-uninformative characters, while the remaining 1558 characters were parsimony-informative. Both the maximum likelihood (ML) and Bayesian Inference (MB) method yielded highly similar trees with almost identical topologies. The phylogenetic relationship was represented using the topological structure obtained from ML analyses, with a final optimization likelihood of -53757.555363 (Fig. 1). The six strains of *Absidia* isolated in this study were divided into three individual branches representing *A.pacifica*, *A.crystalloides*, and *A.pateriformis*. In order to further resolve the three new species and their related species, genetic distances of each DNA marker were calculated and shown in the Suppl. material 1: PDF. The *A.pacifica* is closely related to *A.edaphica* (MLBV = 100, BIPP = 1.00), with genetic distances of 0.739 (ITS), 0.699 (LSU), 0.008 (*Act*), and 0.003 (SSU). The *A.crystalloides* is closely related to *A.oblongispora* and *A.heterospora* (MLBV = 100, BIPP = 1.00), with genetic distances of 0.760 (ITS), 0.716 (LSU), and 0.008 (*Act*) from *A.oblongispora*, and 0.657 (ITS), 0.721 (LSU), and 0.000 (SSU) from *A.heterospora*. The *A.pateriformis* is closely related to *A.jiangxiensis* (MLBV = 100, BIPP = 1.00), with genetic distances of 0.724 (ITS), 0.763 (LSU), 0.002 (TEF-1α), 0.018 (*Act*), and 0.003 (SSU).

### ﻿Taxonomy

The present study reported three novel species, namely *Absidiapacifica* sp. nov., *A.crystalloides* sp. nov., and *A.pateriformis* sp. nov., from soil samples collected in Hainan, China. They are described and illustrated as follows.

#### ﻿ Absidia pacifica

Taxon classificationFungiMucoralesCunninghamellaceae

M.F. Tao, H. Zhao & X.Y. Liu sp. nov.

F9AF049A-B910-530E-B72B-58CD7527AB34

Fungal Names: FN 571915

Fig. 2

##### Type.

China • Hainan Province, Changjiang County, Bawangling National Forest Park (19.08593°N, 109.12275°E), from soil, 14 Oct 2023, M.F. Tao and X.Y. Liu, holotype HMAS 352924, ex-holotype living culture CGMCC3.27497, living cultures XG06955-16 or SAUCC6955-16.

##### Etymology.

The epithet *pacifica* (Lat.) refers to the pacifier-shaped projections.

##### Description.

Mycelia hyaline at first, becoming brownish when mature, aseptate or irregularly septate with age, branched. Stolons hyaline to brownish, smooth, branched, 4.2–8.8 µm in diameter. Rhizoids well-developed, root-like, hyaline, simply branched. Stolons present. Sporangiophores growing from stolons, erect or slightly bent, unbranched, hyaline, single or 2–6 in whorls, 79.1–128.7 µm long, 3.0–6.5 µm wide, sometimes with a swelling beneath sporangia, with a septum 11.3–29.6 µm below apophyses. Apophyses obvious, small, hyaline, slightly pigmented, 4.6–8.4 µm wide at the base and 9.8–20.0 µm wide at the top. Sporangia globose, smooth, dark brown, deliquescent-walled, 23.6–39.2 µm long, 19.0–33.7 µm wide. Collars absent or present. Columellae mostly globose, occasionally oval, 12.8–22.1 µm long, 9.5–21.8 µm wide. Projections pacifier-like, 4.1–7.5 µm long, 1.8–3.9 µm wide. Sporangiospores variously shaped, mostly cylindrical or subglobose, smooth, hyaline, 2.8–6.2 µm long, 2.0–3.9 µm wide. Chlamydospores absent. Zygospores not found.

##### Culture characteristics.

Colonies on PDA at 25 °C for 7 days, reaching 85 mm in diameter, exhibiting an average growth rate of approximately 11.4–12.1 mm/d, white initially, brown with age, irregularly concentrically zonate with ring, flower-shaped, irregularly in reverse, with adjoining satellite colonies at edge.

##### Maximum growth temperature.

35 °C.

##### Additional specimen examined.

China • Hainan Province, Changjiang County, Seven Forks (19.11750°N, 109.15000°E), from soil, 11 Apr 2023, M.F. Tao and X.Y. Liu, living culture XG04136-4 or SAUCC413601; • China, Hainan Province, Changjiang County, Bawangling National Forest Park (19.08593°N, 109.12275°E), from soil, 14 Oct 2023, M.F. Tao and X.Y. Liu, living culture XG06955-15 or SAUCC6955-15.

##### Notes.

Phylogenetically, *A.pacifica* was closely related to *A.edaphica*. Morphologically, the sporangiophores of *A.pacifica* was longer than that of *A.edaphica* (3.0–6.5 µm vs 2.5–5.4 µm), and the distance between the septum and apophysis was shorter in *A.pacifica* than that in *A.edaphica* (11.3–29.6 µm vs 20–33.5 µm). The columellae of *A.pacifica* were larger than those of *A.edaphica* (9.5–21.8 × 12.8–22.1 μm vs 5–9.5 × 6.5–20 μm). Additionally, some columellae in *A.pacifica* lacked a collar structure. The overall length of spores in *A.pacifica* was slightly larger than that in *A.edaphica* (2.8–6.2 µm vs 3.5–5.5 µm), and the shape of sporangiospores in *A.pacifica* was more diverse. Physiologically, the maximum growth temperature for *A.pacifica* was lower than that of *A.edaphica* (35 °C vs 36 °C) (Hurdeal et al. 2021).

**Figure 2.**
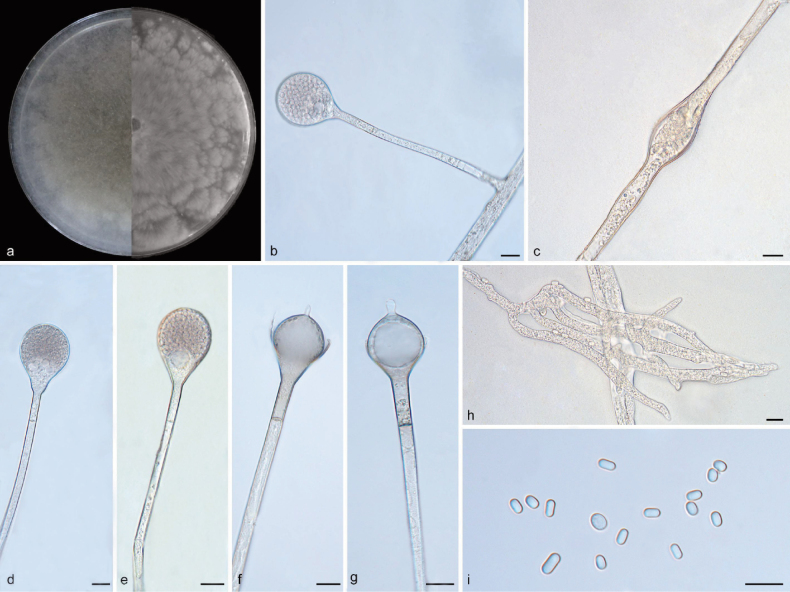
*Absidiapacifica* (holotype: HMAS 352924) **a** obverse and reverse of the culture on PDA **b, d, e** sporangia **c** swellings in hyphae **f, g** columellae **h** rhizoids **i** sporangiospores. Scale bars: 10 μm (**b–i**).

#### ﻿ Absidia crystalloides

Taxon classificationFungiMucoralesCunninghamellaceae

M.F. Tao, H. Zhao & X.Y. Liu sp. nov.

9956DCF8-D112-532A-86CB-65E9938B46EA

Fungal Names: FN 571916

Fig. 3

##### Type.

China • Hainan Province, Changjiang County, Bawangling National Forest Park (19.08593°N, 109.12275°E), from soil, 14 Oct 2023, M.F. Tao and X.Y. Liu, holotype HMAS 352925, ex-holotype living culture CGMCC3.27496, living cultures XG06948-15 or SAUCC6948-15.

##### Etymology.

The epithet *crystalloides* (Lat.) refers to the crystal-like projections.

##### Description.

Mycelia white at first and gradually turning to dark brown, branched, subhyaline to hyaline, aseptate or irregularly septate with age. Rhizoids well-developed, root-like, branched. Stolons hyaline, branched, brownish, smooth, 3.1–7.8 µm in diameter. Sporangiophores growing on stolons, mostly unbranched or simply branched, erect or slightly bent, smooth, single or 2–4 in whorls, 55.8–109.3 µm long, 2.6–4.7 µm wide, with one septum 12.8–24.1 µm below sporangia. Sporangia mainly globose, rarely pyriform, dark brown, smooth, subhyaline, deliquescent-walled, 23.0–28.0 µm long, 23.4–28.0 µm wide. Apophyses distinct, light brown, subhyaline, 3.9–9.4 µm wide at the base and 7.4–17.8 µm wide at the top. Collars clearly present, hyaline. Columellae subglobose to globose, smooth, 9.7–12.6 µm long, 11.7–19.5 µm wide. Projections present or absent, if present, single, crystal-like, 3.1–3.5 µm long, 1.8–2.2 µm wide. Sporangiospores smooth, hyaline, oval or fabiform, exhibiting a slight constriction at the center, 3.1–4.1 µm long, 2.1–2.8 µm wide. Chlamydospores absent. Zygospores not found.

**Figure 3.**
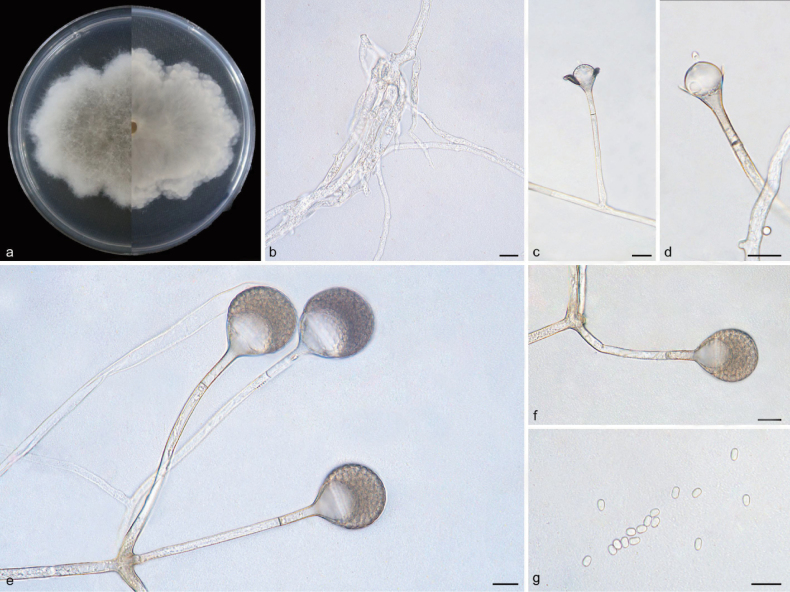
*Absidiacrystalloides* (holotype: HMAS 352925) **a** obverse and reverse of the culture on PDA **b** rhizoids **c, d** columellae **e, f** sporangia **g** sporangiospores. Scale bars: 10 μm (**b–g**).

##### Culture characteristics.

Colonies on PDA at 25 °C for 7 days, reaching 65 mm in diameter, exhibiting an average growth rate of approximately 8.6–9.3 mm/d, white initially, gradually becoming dark brown when mature, irregularly concentrically zonate with ring, irregularly in reverse.

##### Maximum growth temperature.

32 °C.

##### Additional specimen examined.

China • Hainan Province, Changjiang County, Bawangling National Forest Park (19.08593°N, 109.12275°E), from soil, 14 Oct 2023, M.F. Tao and X.Y. Liu, living culture XG06932-1 or SAUCC693201, XG06948-17 or SAUCC6948-17.

##### Notes.

Phylogenetically, *A.crystalloides* was closely related to *A.oblongispora* and *A.heterospora*. Compared with *A.oblongispora*, the *A.crystalloides* exhibited a smaller stolon diameter (3.1–7.8 µm vs 4.0–9.5 µm), the septum was positioned at a greater distance from apophyses (12.8–24.1 µm vs 9.5–16.0 µm), while sporangiophores were located at a shorter distance (55.8–109.3 µm vs 33.0–300.0 µm), apophyses had a wider base width (3.9–9.4 µm vs 3.5–7.5 µm). Physiologically, the maximum growth temperature of *A.crystalloides* was higher (32 °C vs 31 °C). In comparison to *A.heterospora*, the *A.crystalloides* possessed two forms of sporangiospores and had a smaller columella diameter (11.7–19.5 µm vs 10.5–34 µm) (Hesseltine and Ellis 1964; Zhao et al. 2023).

#### ﻿ Absidia pateriformis

Taxon classificationFungiMucoralesCunninghamellaceae

M.F. Tao, H. Zhao & X.Y. Liu sp. nov.

4F7C09DE-C11D-543C-ADB5-8B31A4AD3FC2

Fungal Names: FN 571917

Fig. 4

##### Type.

China • Hainan Province, Ledong County, Jianfengling National Forest Park (18.74540°N, 108.96716°E), from soil, 13 Oct 2023, M.F. Tao and X.Y. Liu, holotype HMAS 352926, ex-holotype living culture CGMCC3.27495, living cultures XG06347-1 or SAUCC6347D-1.

##### Etymology.

The *pateriformis* (Lat.) refers to its bowling-like projections.

##### Description.

Mycelia white at first and gradually turning to light brown, aseptate or septate, branched. Rhizoids well-developed, root-like, simply branched. Stolons hyaline, branched, smooth, 4.4–9.2 µm in diameter. Sporangiophores growing on stolons, erect or slightly bent, mostly unbranched or simply branched, smooth, single or 2–4 in whorls, 33.4–156.5 µm long, 2.7–6.8 µm wide. Sporangia globose to pyriform, smooth, hyaline, deliquescent-walled, 16.8–30.6 µm long, 11.2–26.7 µm wide, and with a septum 9.4–20.6 µm below sporangia. Apophyses obvious, gradually widening from the base to the top, 3.8–11.4 µm wide at the base and 7.1–24.3 µm wide at the top, light brown, hyaline. Collars absent or present. Columellae mostly globose, occasionally oval, 10.9–25.1 µm long, 8.2–21.0 µm wide. Projections obvious, bowling-like, hyaline, single, 3.3–6.7 µm long, 1.2–4.0 µm wide. Sporangiospores smooth, hyaline, oval or globose, 3.1–4.2 µm long, 2.7–4.0 µm wide. Chlamydospores absent. Zygospores not found.

**Figure 4.**
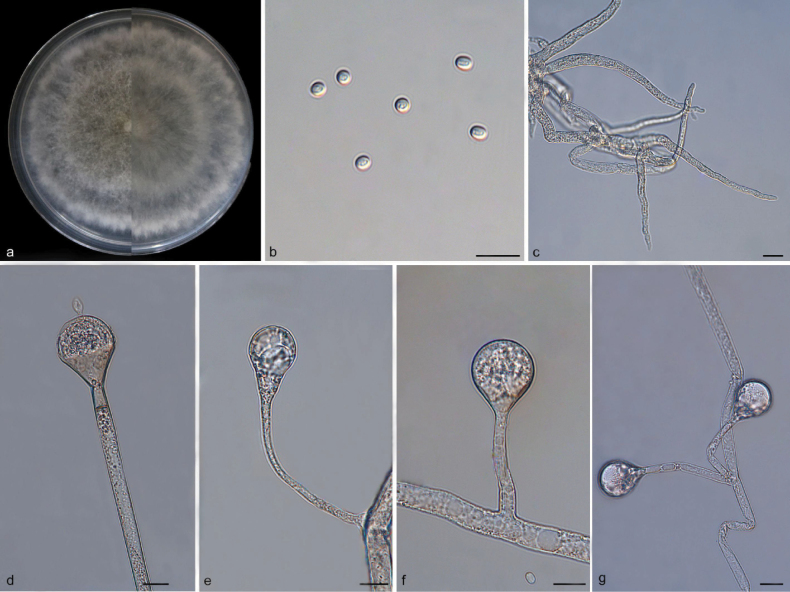
*Absidiapateriformis* (holotype: HMAS 352926) **a** obverse and reverse of the culture on PDA **b** sporangiospores **c** rhizoids **d** columellae **e–g** sporangia. Scale bars: 10 μm (**b–g**).

##### Culture characteristics.

Colonies on PDA at 25 °C for 7 days, reaching 80 mm in diameter, exhibiting an average growth rate of approximately 10.7–11.4 mm/day, initially white, gradually becoming light brown and greenish when mature, regularly in reverse, petaloid at edge.

##### Maximum growth temperature.

30 °C.

##### Additional specimen examined.

China • Hainan Province, Ledong County, Jianfengling National Forest Park (18.74540°N, 108.96716°E), from soil, 13 Oct 2023, M.F. Tao and X.Y. Liu, living culture XG06347D-2, SAUCC634702 or SAUCC6347D-2.

##### Notes.

In the molecular phylogeny, *A.pateriformis* was closely related to *A.jiangxiensis*. Morphologically, the sporangiophores of *A.pateriformis* were at most four-wheeled and branched, while those of *A.jiangxiensis* were at most six-wheeled and unbranched. Additionally, the maximum length of the sporangiophores in *A.jiangxiensis* was significantly greater than that in *A.pateriformis* (156.5 µm vs 280.0 µm). The sporangia size was also smaller in *A.pateriformis* (16.8–30.6 × 11.2–26.7 μm vs 16.5–48 × 16.5–44 μm), with only one projection observed instead of two and a narrower columellae (8–21 µm vs 10–34 µm). Zygospores were not observed in *A.pateriformis* (Zhao et al. 2023).

## ﻿Discussion

*Absidia* is predominantly distributed in soil environments (Richardson 2009). In this study, we investigated the soil in Hainan Province. Based on morphology, growth temperature dynamics and molecular phylogenetic analyses, we identified three novel species in the genus *Absidia*, namely *A.pacifica* sp. nov., *A.crystalloides* sp. nov., and *A.pateriformis* sp. nov. The inclusion of these three taxa altered the topology of the *Absidia* tree. Through joint data analysis of SSU, ITS, LSU, TEF-1α and *Act*, the placement of these new species in the evolutionary tree was robustly supported (*A.pacifica* 100% MLBV and 0.98 BIPP; *A.crystalloides* 100% MLBV and 1.00 BIPP; *A.pateriformis* 100% MLBV and 1.00 BIPP; Fig. 1). The phylogenetic analysis also revealed a close relationship between *A.pacifica* and *A.edaphica* (100% MLBV, 1.00 BIPP), whereas *A.crystalloides* exhibited a close affinity to both *A.oblongispora* and *A.heterospora* (100% MLBV, 1.00 BIPP), and *A.pateriformis* demonstrated a strong association with *A.jiangxiensis* (100% MLBV, 1.00 BIPP). Simultaneously, we observed certain variations in morphological structure and physiology, which served as the basis for species identification. The three newly discovered species and their related counterparts exhibited varying degrees of discrepancy in sporangiophores, sporangia, projections, sporangiospores, and other structures. Furthermore, the maximum growth temperatures were found to be distinguishable among these species. These dissimilarities served as the foundation for the identification of novel species.

Currently, the phylogenetic analysis of *Absidia* primarily employed a combined analysis of SSU, ITS, LSU, TEF-1α, and *Act.* Previous studies demonstrated that due to the significant variation among *Absidia* species, relying solely on ITS for identifying *Absidia* species might yield inaccurate results; therefore, it was essential to incorporate LSU or other gene markers in the analysis process. Nucleotide sequences of SSUITS, LSU, TEF-1α, and *Act* fragments served as robust tools for *Absidia* identification (White et al. 1990; Hoffmann et al. 2007, 2013; Ariyawansa et al. 2015).

Early on, *Lichtheimia* was initially classified as *Absidia* due to its morphological resemblance, Hoffmann et al. (2007) categorized *Absidia* into three groups based on their maximum growth temperature: thermotolerant (≥37 °C), mesophilic (25 °C–34 °C), and mycoparasitic (≤30 °C). The thermotolerant strains were subsequently reclassified as *Lichtheimia*. Currently, there were hardly any identified strains of *Absidia* capable of growing at a maximum temperature of 37 °C (Zhao et al. 2022b, 2023). This study further confirmed this conclusion by demonstrating that the maximum growth temperatures for *A.pacifica*, *A.crystalloides*, and *A.pateriformis* were 35 °C, 32 °C and 30 °C, respectively.

In China, the majority of *Absidia* species were predominantly distributed in Xinjiang, Taiwan, and Yunnan, primarily inhabiting tropical, subtropical, and temperate regions (Zhao et al. 2022b). However, due to its limited tolerance for high temperatures, most *Absidia* species found in tropical areas were typically restricted to cool and humid environments such as forest soil and dung (Cordeiro et al. 2020; Lima et al. 2021). The discovery of the three new species in this study was exclusively made in the tropical forest soil of Hainan Island. This finding further validated the remarkable species diversity of *Absidia* within tropical and subtropical regions while also providing valuable insights for future exploration into additional *Absidia* strains.

## Supplementary Material

XML Treatment for Absidia pacifica

XML Treatment for Absidia crystalloides

XML Treatment for Absidia pateriformis

## ﻿Additional information

### Author ORCIDs

Meng-Fei Tao https://orcid.org/0009-0008-9053-1310

Zi-Ying Ding https://orcid.org/0009-0003-1618-5740

Yi-Xin Wang https://orcid.org/0009-0001-5231-914X

Zhao-Xue Zhang https://orcid.org/0000-0002-4824-9716

Heng Zhao https://orcid.org/0000-0003-2938-5613

Xiao-Yong Liu https://orcid.org/0000-0002-8808-010X

### Data availability

All of the data that support the findings of this study are available in the main text or Supplementary Information.

## Supplementary materials

Supplementary material 1 Estimates of evolutionary divergence between sequence based on ITS, LSU, TEF, ACT, SSU Data type pdf Explanation note Estimates of evolutionary divergence between sequence based on ITS, LSU, TEF, ACT and SSU. Three new species and their relatives were selected to study, and the number of base differences per site from between sequences are shown. Standard error estimate(s) are shown above the diagonal. Evolutionary analyses were conducted in MEGA7. https://binary.pensoft.net/file/1178855This dataset is made available under the Open Database License (http://opendatacommons.org/licenses/odbl/1.0/). The Open Database License (ODbL) is a license agreement intended to allow users to freely share, modify, and use this Dataset while maintaining this same freedom for others, provided that the original source and author(s) are credited.Meng-Fei Tao

Supplementary material 2 The combined ITS + LSU + TEF + ACT + SSU sequence matrix used in this study Data type fas Explanation note Phylogenetic analyses of SSU, ITS, LSU rDNA, TEF-1α and Act were conducted for a total of 91 sequences, composing 36 species of Absidia and two outgroups, Cunninghamellablakesleeana (CBS 782.68) and C.elegans (CBS 167.53). The phylogenetic analysis encompassed a total of 4,785 characters, with 516 of SSU, 996 of ITS, 1,874 of LSU, 743 of TEF-1α, 660 of Act characters. https://binary.pensoft.net/file/1178856This dataset is made available under the Open Database License (http://opendatacommons.org/licenses/odbl/1.0/). The Open Database License (ODbL) is a license agreement intended to allow users to freely share, modify, and use this Dataset while maintaining this same freedom for others, provided that the original source and author(s) are credited.Meng-Fei Tao

## References

[B1] Ariyawansa HA Hyde KD Jayasiri SC Buyck B Chethana KWT Dai DQ Dai YC Daranagama DA Jayawardena RS Lücking R Ghobad-Nejhad M Niskanen T Thambugala KM Voigt K Zhao RL Li G-J Doilom M Boonmee S Yang ZL Cai Q Cui Y-Y Bahkali AH Chen J Cui BK Chen JJ Dayarathne MC Dissanayake AJ Ekanayaka AH Hashimoto A Hongsanan S Jones EBG Larsson E Li WJ Li Q-R Liu JK Luo ZL Maharachchikumbura SSN Mapook A McKenzie EHC Norphanphoun C Konta S Pang KL Perera RH Phookamsak R Phukhamsakda C Pinruan U Randrianjohany E Singtripop C Tanaka K Tian CM Tibpromma S Abdel-Wahab MA Wanasinghe DN Wijayawardene NN Zhang J-F Zhang H Abdel-Aziz FA Wedin M Westberg M Ammirati JF Bulgakov TS Lima DX Callaghan TM Callac P Chang C-H Coca LF Dal-Forno M Dollhofer V Fliegerová K Greiner K Griffith GW Ho H-M Hofstetter V Jeewon R Kang JC Wen T-C Kirk PM Kytövuori I Lawrey JD Xing J Li H Liu ZY Liu XZ Liimatainen K Lumbsch HT Matsumura M Moncada B Nuankaew S Parnmen S de Azevedo Santiago ALCM Sommai S Song Y de Souza CAF de Souza-Motta CM Su HY Suetrong S Wang Y Wei S-F Wen TC Yuan HS Zhou LW Réblová M Fournier J Camporesi E Luangsa-ard JJ Tasanathai K Khonsanit A Thanakitpipattana D Somrithipol S Diederich P Millanes AM Common RS Stadler M Yan JY Li XH Lee HW Nguyen TTT Lee HB Battistin E Marsico O Vizzini A Vila J Ercole E Eberhardt U Simonini G Wen H-A Chen X-H Miettinen O Spirin V (2015) Fungal diversity notes 111–252: Taxonomic and phylogenetic contributions to fungal taxa. Fungal Diversity 75(1): 27–274. 10.1007/s13225-015-0346-5

[B2] Ashton H (2009) Ainsworth and Bisby’s Dictionary of the Fungi (10^th^ edn). Reference Reviews 23(5): 42–42. 10.1108/09504120910969104

[B3] Benny GL Humber RA Morton JB (2001) Zygomycota: Zygomycetes. In: McLaughlin DJ McLaughlin EG Lemke PA (Eds) Systematics and Evolution, 1st edn.Springer, Berlin, Heidelberg, 113–146. 10.1007/978-3-662-10376-0_6

[B4] Constantinides J Misra A Nassab R Wilson Y (2008) *Absidiacorymbifera* fungal infection in burns: A case report and review of the literature. Journal of Burn Care & Research; Official Publication of the American Burn Association 29(2): 416–419. 10.1097/BCR.0b013e318166da78 18354306

[B5] Cordeiro TR Nguyen TT Lima DX Silva SC Lima CL Leitão JD Gurgel L Lee HB Santiago AL (2020) Two new species of the industrially relevant genus *Absidia* (Mucorales) from soil of the Brazilian Atlantic Forest. Acta Botanica Brasílica 34(3): 549–558. 10.1590/0102-33062020abb0040

[B6] Doyle JJ Doyle JL (1990) Isolation of plant DNA from fresh tissue. Focus (San Francisco, Calif.) 12: 13–15. 10.2307/2419362

[B7] Guo LD Hyde KD Liew ECY (2000) Identification of endophytic fungi from Livistona chinensis based on morphology and rDNA sequences. The New Phytologist 147(3): 617–630. 10.1046/j.1469-8137.2000.00716.x 33862946

[B8] Hesseltine CW Ellis JJ (1964) The Genus *Absidia*: *Gongronella* and cylindrical-spored species of *Absidia.* Mycologia 56(4): 568–601. 10.1080/00275514.1964.12018145

[B9] Hoffmann K (2010) Identification of the genus *Absidia* (Mucorales, Zygomycetes): a comprehensive taxonomic revision. In: Gherbawy Y Voigt K (Eds) Molecular Identification of Fungi, 1st, edn.Springer, Berlin, 439–460. 10.1007/978-3-642-05042-8-19

[B10] Hoffmann K Voigt K (2009) *Absidiaparricida* plays a dominant role in biotrophic fusion parasitism among mucoralean fungi (Zygomycetes): *Lentamyces*, a new genus for *A.parricida* and *A.zychae*. Plant Biology 11(4): 537–554. 10.1111/j.1438-8677.2008.00145.x 19538392

[B11] Hoffmann K Discher S Voigt K (2007) Revision of the genus *Absidia* (Mucorales, Zygomycetes) based on physiological, phylogenetic, and morphological characters; thermotolerant *Absidia* spp. form a coherent group Mycocladiaceae fam. nov. Mycological Research 111(10): 1169–1183. 10.1016/j.mycres.2007.07.002 17997297

[B12] Hoffmann K Pawłowska J Walther G Wrzosek M de Hoog GS Benny GL Kirk PM Voigt K (2013) The family structure of the Mucorales: A synoptic revision based on comprehensive multigene-genealogies. Persoonia 30(1): 57–76. 10.3767/003158513X666259 24027347 PMC3734967

[B13] Huelsenbeck JP Ronquist F (2001) MRBAYES: Bayesian inference of phylogenetic trees. Bioinformatics (Oxford, England) 17(8): 754–755. 10.1093/bioinformatics/17.8.754 11524383

[B14] Hurdeal V G Gentekaki E Lee H B Jeewon R Hyde K D Tibpromma S Mortimer P E Xu J (2021) Mucoralean fungi in Thailand: novel species of *Absidia* from tropical forest soil. Cryptogamie Mycol 42: 39–61. 10.5252/CRYPTOGAMIE-MYCOLOGIE2021V42A4

[B15] Hurdeal VG Jones EBG Gentekaki E (2023) *Absidia*zygospora (Mucoromycetes), a new species from nan province, Thailand. Studies in Fungi 8(1): 0. 10.48130/SIF-2023-0015

[B16] Jaklitsch WM Komon M Kubicek CP Druzhinina IS (2005) *Hypocreavoglmayrii* sp. nov. from the Austrian Alps represents a new phylogenetic clade in *Hypocrea*/*Trichoderma*. Mycologia 97(6): 1365–1378. 10.1080/15572536.2006.11832743 16722227

[B17] Kristanti RA Fikri Ahmad Zubir MM Hadibarata T (2016) Biotransformation studies of cresol red by *Absidiaspinosa* M15. Journal of Environmental Management 172: 107–111. 10.1016/j.jenvman.2015.11.017 26922501

[B18] Kumar S Stecher G Tamura K (2016) MEGA7: Molecular evolutionary genetics analysis version 7.0 for bigger datasets. Molecular Biology and Evolution 33(7): 1870–1874. 10.1093/molbev/msw054 27004904 PMC8210823

[B19] Lima DX Cordeiro TR de Souza CA de Oliveira R Lee HB Souza-Motta CM Santiago AL (2020) Morphological and molecular evidence for two new species of *Absidia* from neotropic soil. Phytotaxa 446(1): 61–71. 10.11646/phytotaxa.446.1.8

[B20] Lima CL Lima DX Cordeiro TR Lee HB Nguyen TT Gurgel L Santiago AL (2021) *Absidiabonitoensis* (Mucorales, Mucoromycota), a new species isolated from the soil of an upland Atlantic Forest in Northeastern Brazil. Nova Hedwigia 112(1–2): 241–251. 10.1127/nova_hedwigia/2021/0614

[B21] Mackenzie DW Soothill JF Millar JH (1988) Meningitis caused by *Absidiacorymbifera.* The Journal of Infection 17(3): 241–248. 10.1016/S0163-4453(88)96570-X 3216134

[B22] Miller MA Pfeiffer W Schwartz T (2010) Creating the CIPRES Science Gateway for inference of large phylogenetic trees. 2010 Gateway Computing Environments Workshop (GCE), 1–8. 10.1109/GCE.2010.5676129

[B23] Nylander J A A (2004) MrModeltest v2. [Program distributed by the author] http://www.abc.se/–nylander/mrmodeltest2/mrmodeltest2.html

[B24] Rehner SA Buckley E (2005) A Beauveria phylogeny inferred from nuclear ITS and EF1-α sequences: Evidence for cryptic diversification and links to Cordyceps teleomorphs. Mycologia 97(1): 84–98. 10.3852/mycologia.97.1.84 16389960

[B25] Richardson M (2009) The ecology of the Zygomycetes and its impact on environmental exposure. Clinical Microbiology and Infection 15(5): 2–9. 10.1111/j.1469-0691.2009.02972.x 19754749

[B26] Sordon S Popłoński J Tronina T Huszcza E (2019) Regioselective O-glycosylation of flavonoids by fungi Beauveria bassiana, *Absidiacoerulea* and *Absidiaglauca.* Bioorganic Chemistry 93: 102750. 10.1016/j.bioorg.2019.01.046 30755333

[B27] Stamatakis A (2014) RAxML version 8: A tool for phylogenetic analysis and post-analysis of large phylogenies. Bioinformatics (Oxford, England) 30(9): 1312–1313. 10.1093/bioinformatics/btu033 24451623 PMC3998144

[B28] Swofford D L (2002) PAUP*. Phylogenetic analysis using parsimony (*and other methods). Version 4.0b10. 10.1111/j.0014-3820.2002.tb00191.x

[B29] van Tieghem P (1876) Troisieme memoire sur les Mucorinees. Ann Sci Nat Bot Ser 6(4): 312–398.

[B30] Varona DM Sánchez JC Muñoz LA Cantos EM Moraleda LR (2015) Keratitis caused by *Absidiacorymbifera* in an immunocompetent male with no corneal injuries. Archivos de la Sociedad Española de Oftalmología 90(3): 139–141. 10.1016/j.oftal.2014.02.020 25443187

[B31] Voigt K Wöstemeyer J (2000) Reliable amplification of actin genes facilitates deep-level phylogeny. Microbiological Research 155(3): 179–195. 10.1016/S0944-5013(00)80031-2 11061186

[B32] von Arx JA (1982) On Mucoraceae s. str. and other families of the *Mucorales.* Sydowia 35: 10–26.

[B33] White TJ Bruns T Lee S Taylor J (1990) Amplification and direct sequencing of fungal ribosomal RNA genes for phylogenetics. In: Innis MA, Gelfand D, Sninsky J et al. (Eds) PCR Protocols: A Guide to Methods and Applications. Academic Press, New York, USA, 315–322. 10.1016/B978-0-12-372180-8.50042-1

[B34] Zhang TY Yu Y Zhu H Yang SZ Yang TM Zhang MY Zhang YX (2018) *Absidiapanacisoli* sp. nov., isolated from rhizosphere of Panax notoginseng. International Journal of Systematic and Evolutionary Microbiology 68(8): 2468–2472. 10.1099/ijsem.0.002857 29927367

[B35] Zhang Z Liu R Liu S Mu T Zhang X Xia J (2021) Morphological and phylogenetic analyses reveal two new species of Sporocadaceae from Hainan, China. MycoKeys 88: 171–192. 10.3897/mycokeys.88.82229 PMC9023435 35585932

[B36] Zhao H Nie Y Zong T Dai Y Liu X (2022a) Three new species of *Absidia* (Mucoromycota) from China based on phylogeny, morphology and physiology. Diversity 14(2): 132. 10.3390/d14020132

[B37] Zhao H Nie Y Zong T Wang Y Wang M Dai Y Liu X (2022b) Species diversity and ecological habitat of *Absidia* (Cunninghamellaceae, Mucorales) with emphasis on five new species from forest and grassland soil in China. Journal of Fungi (Basel, Switzerland) 8(5): 471. 10.3390/jof8050471 35628728 PMC9146633

[B38] Zhao H Nie Y Zong T Wang K Lv M Cui Y Tohtirjap A Chen J Zhao C Wu F Cui B Yuan Y Dai Y Liu X (2023) Species diversity, updated classification and divergence times of the phylum Mucoromycota. Fungal Diversity 123(1): 49–157. 10.1007/s13225-023-00525-4

[B39] Zheng RY Liu XY (2009) Taxa of *Pilaira* (Mucorales, Zygomycota) from China. Nova Hedwigia 88(1–2): 255–267. 10.1127/0029-5035/2009/0088-0255

[B40] Zheng R Y Chen G Q (2007) A monograph of *Rhizopus*. Sydowia 59: 273.

[B41] Zheng RY Liu XY Li RY (2009) More *Rhizomucor* causing human mucormycosis from China: *R.chlamydosporus* sp. nov. Sydowia 61: 135–147.

[B42] Zong TK Zhao H Liu XL Ren LY Zhao CL Liu XY (2021) Taxonomy and phylogeny of four new species in *Absidia* (Cunninghamellaceae, Mucorales) from China. Frontiers in Microbiology 12: 677836. 10.3389/fmicb.2021.677836 PMC8371387 34421840

