# Supplementary material for: ﻿Unveiling species diversity within early-diverging fungi from China II: Three new species of Absidia (Cunninghamellaceae, Mucoromycota) from Hainan Province
Source: MycoKeys. 2024 Nov 20;110:255–72. doi: 10.3897/mycokeys.110.129120 (PMC11602966; doi:10.3897/mycokeys.110.129120)
Supplement: Supplementary material 1 — Estimates of evolutionary divergence between sequence based on ITS, LSU, TEF, ACT, SSU [file mycokeys-110-255-s001.pdf]

Supplementary Material

Table S1. Estimates of Evolutionary Divergence between Sequences based on ITS

A. pateriformis SAUCC 634702		0.000	0.019	0.019	0.020	0.020	0.021	0.020	0.021	0.020
A. pateriformis CGMCC 3.27495	0.000		0.019	0.019	0.020	0.020	0.021	0.020	0.021	0.020
A. crystalloides CGMCC 3.27496	0.765	0.765		0.000	0.020	0.020	0.020	0.019	0.020	0.021
A. crystalloides SAUCC 693201	0.765	0.765	0.000		0.020	0.020	0.020	0.019	0.020	0.021
A. pacifica CGMCC 3.27497	0.730	0.730	0.747	0.747		0.000	0.020	0.020	0.019	0.022
A. pacifica SAUCC 413601	0.730	0.730	0.747	0.747	0.000		0.020	0.020	0.019	0.022
A. edaphica MFLUCC 20-0088	0.719	0.719	0.741	0.741	0.739	0.739		0.022	0.020	0.021
A. jiangxiensis CGMCC 3.16105	0.724	0.724	0.745	0.745	0.739	0.739	0.724		0.019	0.021
A. oblongispora CGMCC 3.16061	0.711	0.711	0.760	0.760	0.741	0.741	0.760	0.741		0.020
A. heterospora SHTH021	0.745	0.745	0.657	0.657	0.475	0.475	0.745	0.708	0.737	

Notes: The number of base differences per site from between sequences are shown. Standard error estimate(s) are shown above the diagonal. The analysis involved 10 nucleotide sequences. All positions containing gaps and missing data were eliminated. There were a total of 463 positions in the final dataset. Evolutionary analyses were conducted in MEGA7.

Table S2. Estimates of Evolutionary Divergence between Sequences based on LSU

A. pateriformis SAUCC 634702		0.000	0.019	0.019	0.015	0.015	0.017	0.016	0.016	0.017
A. pateriformis CGMCC 3.27495	0.000		0.019	0.019	0.015	0.015	0.017	0.016	0.016	0.017
A. crystalloides CGMCC 3.27496	0.616	0.616		0.000	0.018	0.018	0.017	0.016	0.017	0.015
A. *crystalloides* SAUCC 693201	0.616	0.616	0.000		0.018	0.018	0.017	0.016	0.017	0.015
A. pacifica CGMCC 3.27497	0.206	0.206	0.594	0.594		0.000	0.017	0.016	0.016	0.017
A. pacifica SAUCC 413601	0.206	0.206	0.594	0.594	0.000		0.017	0.016	0.016	0.017
A. edaphica MFLUCC 20-0088	0.703	0.703	0.725	0.725	0.699	0.699		0.015	0.016	0.017
A. jiangxiensis CGMCC 3.16105	0.763	0.763	0.761	0.761	0.752	0.752	0.776		0.016	0.015
A. oblongispora CGMCC 3.16061	0.752	0.752	0.716	0.716	0.772	0.772	0.731	0.742		0.018
A. heterospora SHTH021	0.740	0.740	0.721	0.721	0.748	0.748	0.731	0.775	0.728	

Notes: The number of base differences per site from between sequences are shown. Standard error estimate(s) are shown above the diagonal. The analysis involved 10 nucleotide sequences. All positions containing gaps and missing data were eliminated. There were a total of 670 positions in the final dataset. Evolutionary analyses were conducted in MEGA7.

Table S3. Estimates of Evolutionary Divergence between Sequences based on TEF-1α

A. pateriformis SAUCC 634702		0.000	0.006	0.006	0.006	0.006	0.002
A. pateriformis CGMCC 3.27495	0.000		0.006	0.006	0.006	0.006	0.002
A. crystalloides CGMCC 3.27496	0.028	0.028		0.000	0.006	0.006	0.006
A. crystalloides SAUCC 693201	0.028	0.028	0.000		0.006	0.006	0.006
A. pacifica CGMCC 3.27497	0.031	0.031	0.029	0.029		0.000	0.006
A. pacifica SAUCC 413601	0.031	0.031	0.029	0.029	0.000		0.006
A. jiangxiensis CGMCC 3.16105	0.002	0.002	0.029	0.029	0.032	0.032	

Notes: The number of base differences per site from between sequences are shown. Standard error estimate(s) are shown above the diagonal. The analysis involved 7 nucleotide sequences. Codon positions included were 1st+2nd+3rd+Noncoding. All positions containing gaps and missing data were eliminated. There were a total of 653 positions in the final dataset. Evolutionary analyses were conducted in MEGA7.

Table S4. Estimates of Evolutionary Divergence between Sequences based on *Act*

A. pateriformis SAUCC 634702		0.000	0.010	0.010	0.011	0.011	0.011	0.006	0.010
A. pateriformis CGMCC 3.27495	0.000		0.010	0.010	0.011	0.011	0.011	0.006	0.010
A. crystalloides CGMCC 3.27496	0.065	0.065		0.000	0.011	0.011	0.011	0.010	0.004
A. crystalloides SAUCC 693201	0.065	0.065	0.000		0.011	0.011	0.011	0.010	0.004
A. pacifica CGMCC 3.27497	0.087	0.087	0.073	0.073		0.000	0.004	0.012	0.011
A. pacifica SAUCC 413601	0.087	0.087	0.073	0.073	0.000		0.004	0.012	0.011
A. edaphica MFLUCC 20-0088	0.087	0.087	0.073	0.073	0.008	0.008		0.011	0.011
A. jiangxiensis CGMCC 3.16105	0.018	0.018	0.065	0.065	0.089	0.089	0.089		0.010
A. oblongispora CGMCC 3.16061	0.065	0.065	0.008	0.008	0.073	0.073	0.073	0.065	

Notes: The number of base differences per site from between sequences are shown. Standard error estimate(s) are shown above the diagonal. The analysis involved 9 nucleotide sequences. Codon positions included were 1st+2nd+3rd+Noncoding. All positions containing gaps and missing data were eliminated. There were a total of 507 positions in the final dataset. Evolutionary analyses were conducted in MEGA7.

Table S5. Estimates of Evolutionary Divergence between Sequences based on SSU

A. pateriformis SAUCC 634702		0.000	0.008	0.008	0.007	0.007	0.007	0.002	0.008
A. pateriformis CGMCC 3.27495	0.000		0.008	0.008	0.007	0.007	0.007	0.002	0.008
A. crystalloides CGMCC 3.27496	0.050	0.050		0.000	0.007	0.007	0.007	0.008	0.000
A. crystalloides SAUCC 693201	0.050	0.050	0.000		0.007	0.007	0.007	0.008	0.000
A. pacifica CGMCC 3.27497	0.043	0.043	0.038	0.038		0.000	0.002	0.007	0.007
A. pacifica SAUCC 413601	0.043	0.043	0.038	0.038	0.000		0.002	0.007	0.007
A. edaphica MFLUCC 20-0088	0.043	0.043	0.038	0.038	0.003	0.003		0.007	0.007
A. jiangxiensis CGMCC 3.16105	0.003	0.003	0.050	0.050	0.046	0.046	0.046		0.008
A. heterospora SHTH021	0.050	0.050	0.000	0.000	0.038	0.038	0.038	0.050	

Notes: The number of base differences per site from between sequences are shown. Standard error estimate(s) are shown above the diagonal. The analysis involved 9 nucleotide sequences. All positions containing gaps and missing data were eliminated. There were a total of 736 positions in the final dataset. Evolutionary analyses wen MEGA7.
